# Tryptophan oxidation in young children with environmental enteric dysfunction classified by the lactulose rhamnose ratio

**DOI:** 10.1093/ajcn/nqac171

**Published:** 2022-06-14

**Authors:** Nirupama Shivakumar, Jean W Hsu, Sindhu Kashyap, Tinku Thomas, Anura V Kurpad, Farook Jahoor

**Affiliations:** Division of Nutrition, St. John's Research Institute, St. John's National Academy of Health Sciences, Bangalore, India; USDA/Agricultural Research Service, Children's Nutrition Research Center, Department of Pediatrics, Baylor College of Medicine, Houston, TX, USA; Division of Nutrition, St. John's Research Institute, St. John's National Academy of Health Sciences, Bangalore, India; Department of Biostatistics, St. John's Medical College, St. John's National Academy of Health Sciences, Bangalore, India; Division of Nutrition, St. John's Research Institute, St. John's National Academy of Health Sciences, Bangalore, India; Department of Physiology, St. John's Medical College, St. John's National Academy of Health Sciences, Bangalore, India; USDA/Agricultural Research Service, Children's Nutrition Research Center, Department of Pediatrics, Baylor College of Medicine, Houston, TX, USA

**Keywords:** environmental enteric dysfunction, children, lactulose rhamnose ratio, kynurenine tryptophan ratio, tryptophan oxidation, tryptophan flux

## Abstract

**Background:**

In young children, associations between linear growth faltering, environmental enteric dysfunction (EED), and the plasma kynurenine (Kyn)/tryptophan (Trp) ratio (KTR) have led to the proposal that higher Trp catabolism in response to intestinal/systemic inflammation limits Trp availability for protein synthesis, resulting in impaired growth.

**Objectives:**

We sought to estimate the Trp oxidation rate and the Trp conversion rate to Kyn in young children with and without EED.

**Methods:**

Children aged 18–24 mo, from urban slums, were assigned to EED (*n* = 19) or no-EED (*n* = 26) groups on the basis of a urinary lactulose/rhamnose ratio (LRR) cutoff based on mean + 2 SDs of LRR (≥0.068) in normal age- and sex-matched, high–socioeconomic status children. Plasma KTR and fecal biomarkers of EED were measured. Trp oxidation in the fed state was measured using ^13^C_1_-Trp in an oral plateau feeding protocol.

**Results:**

The median (quartile 1, quartile 3) fasted KTR was 0.089 (0.066, 0.110) in children with EED compared with 0.070 (0.050, 0.093) in children with no EED (*P* = 0.077). However, there was no difference in fed-state Trp oxidation [median (quartile 1, quartile 3) 3.1 (1.3, 5.8) compared with 3.9 (1.8, 6.0) µmol/kg FFM/h, respectively, *P* = 0.617] or Trp availability for protein synthesis [42.6 (36.5, 45.7) compared with 42.5 (37.9, 46.9) µmol/kg FFM/h, respectively, *P* = 0.868] between the groups. In contrast, the median (quartile 1, quartile 3) fractional synthesis rates of Kyn [12.5 (5.4, 20.0) compared with 21.3 (16.1, 24.7) %pool/h, *P* = 0.005] and the fraction of Ala derived from Trp [0.007 (0.005, 0.015) compared with 0.012 (0.008, 0.018), *P* = 0.037], respectively, in the plasma compartment were significantly slower in the EED group. Fecal biomarkers of EED did not differ between the groups.

**Conclusions:**

The static plasma KTR value is not a good indicator of the dynamic Trp flux down its oxidative pathway. In a poor sanitary environment, children without EED actually have a faster Kyn synthesis rate, which might be beneficial, because of the cytoprotective and anti-inflammatory functions of downstream metabolites. This study was registered in the Clinical Trials Registry of India as CTRI/2017/02/007921.

## Introduction

The UNICEF 2020 joint global malnutrition report documents a high prevalence (21%) of stunting in children < 5 y of age, with children in India having an even higher prevalence of 35% (1, 2). Stunting, in the long term, is associated with increased morbidity, poor cognitive development and school performance, and reduced economic productivity, which could even trigger intergenerational consequences (3). In recent years, environmental enteropathy (EE) or environmental enteric dysfunction (EED), which could affect nutrient absorption, has gained recognition as one possible etiology for stunting, supported by studies that demonstrated poor growth responses to feeding trials in low- and -middle income countries (LMIC) (3–6). EED is an acquired subclinical disorder of the small intestine in response to chronic fecal–oral pathogenic exposure, with reduced digestion and absorption of nutrients (5, 7–10).

Tryptophan (Trp), an indispensable dietary amino acid (AA), has been explored as a growth supplement in animals and children, partly due to the immunomodulatory and cytoprotective functions of its metabolites (11–13). The impact of sanitary conditions on Trp availability has been demonstrated in animal studies in which pigs raised in poor sanitary conditions that resulted in moderate systemic inflammation had lower plasma Trp concentrations due to increased Trp oxidation (14). The potential implications of this relation extend further in studies from Bangladesh, which suggest an association between increased Trp oxidation in young children and linear growth faltering based on moderate associations between plasma Trp and kynurenine (Kyn) concentrations and Kyn/Trp ratio (KTR) with linear growth (15, 16). An increased KTR in this setting was possibly indicative of an increased flux of Trp through the Kyn-NAD pathway by the immune response–activated indolamine 2,3-dioxygenase (IDO1) enzyme (17). Thus, it was hypothesized that in young children with EED, because of local (intestinal) and/or systemic inflammatory activation, there is accelerated catabolism of dietary Trp by IDO1, leading to a limitation of Trp for net protein synthesis and hence impaired growth (15, 16), but this has not been measured. There are also several other factors that influence the KTR, including Trp catabolism through the induction of hepatic Trp 2,3, dioxygenase (TDO) by substrate availability, feedback inhibition by reduced NAD(P), glucocorticoids, high plasma leucine concentration, and variable plasma Kyn concentration influenced by its metabolism, vitamin B6 status, and renal clearance (17, 18).

The primary objective of this study was to test the hypothesis that children with EED, compared with those without EED (no-EED group), would have a higher Trp conversion rate to Kyn or its downstream metabolite alanine, and hence, a higher oxidation rate leading to lower availability for whole-body protein synthesis. Steady-state Trp kinetics were measured using oral L-[1-^13^C]Trp (19) in the fed state. Since EED lacks a clinical definition or diagnostic criteria using biomarker cutoffs, the lactulose/rhamnose ratio (LRR) was used to sort children into EED (high LRR) and no EED groups. In addition, associations between Trp oxidation and potential modulatory factors such as nutrient intake, plasma amino acid and Kyn concentrations, total energy expenditure, and fecal and plasma inflammatory biomarkers were evaluated.

## Participants and Methods

### Participants

Healthy children (*n* = 20) from a high–socioeconomic status (SES) community, based on the Kuppuswamy classification (20), were recruited from the pediatric well-baby clinic of St. John's Hospital after undergoing screening for inclusion and exclusion criteria. These children only participated in the intestinal permeability test to provide normal values to be used as the basis for identifying apparently healthy children in the population. Children aged 18–24 mo of either sex who were no longer breastfeeding at the time of recruitment were eligible for participation and recruited from urban slums near St. John's Medical College and Hospital, Bengaluru, India. Those with chronic illness (medical or surgical), diagnosed with malabsorption syndromes, congenital diseases, history of diarrhea or vomiting, or usage of antibiotics in the past 4 wk were excluded. The chronic illnesses in children who were excluded were congenital cardiac disease, hyperactive airway disease or inborn errors of metabolism, or diagnosed and/or treated for tuberculosis in the previous 1 y. Children were also excluded if they had malabsorption syndromes that were clinically investigated and diagnosed, such as cystic fibrosis, Shwachman-Diamond syndrome, lactose intolerance, cow's milk or other protein-sensitive enteropathies, celiac disease, acute or chronic pancreatitis, liver or biliary tract disorders, congenital bowel mucosal defects, surgical bowel resections/short-bowel syndrome, and Crohns’ disease. Participants also underwent a thorough physical examination by a pediatrician to rule out any of these exclusion conditions. The participant screening and enrollment details are provided in **Supplemental Figure 1**. The study was approved by the Institutional Ethical Review Board of St. John's Medical College and Hospital and by the Institutional Review Board for Human Subject Research of Baylor College of Medicine & Affiliated Hospitals. Written informed consent was obtained from the primary caregivers of the participants.

Field staff conducted interviews with the primary caregivers using structured questionnaires to obtain information on family structure, household characteristics, childhood morbidity, immunization history, and infant and young child feeding practices. On 4 nonconsecutive days (3 weekdays, 1 weekend), 24-h diet recall data were collected to determine the dietary intake of the participants. Anthropometric measurements were performed in triplicates, using standard procedures and equipment; weight was measured to the nearest 10 g (Seca 354) and length to the nearest millimeter (Seca 417). An average of 3 values was used for analysis. The nutritional status indicators weight-for-age *z*-scores (WAZ), length-for-age *z*-scores (LAZ), and weight-for-length *z*-scores (WLZ) were obtained using the WHO anthroplus software (version 3.2.2, January 2011).

### Screening for intestinal permeability

Children were screened for the presence of impaired intestinal permeability with the dual sugar absorption test, using lactulose (L) and rhamnose (R) sugars (henceforth referred to as the LR protocol). A short-duration (2 h) LR protocol (21,22) was adopted and used during screening as follows. Mothers were trained to collect the early morning urine void into a sterile container, which served as a basal sample for the experiment. The children were fed an early breakfast and brought to the research facility, where they were encouraged to void again before consuming 14 mL of a solution of 1.3 g L and 0.3 g R (Tokyo Chemical Industry) at 2 h postprandially. The study children were encouraged to drink water (but no other liquids), and no food was provided for the next 2 h. During this 2-h period, all urine voids were collected through a preweighed plastic diaper lined with a sterile cotton pad. If no urine was collected at the end of 2 h, the collection period was continued for an additional 0.5 h. The diapers were immediately weighed to quantify urine volume, and the sample was extracted and stored on ice until the end of the experiment, when the samples were proportionally pooled (per volume voided), divided into aliquots, and placed into sterile cryovials, and stored at −20°C until analysis. Urinary L and R concentrations were measured by an isotope dilution method modified from a previously described procedure (23). Briefly, a weighed volume of urine sample was spiked with a known quantity (5 nmol) of U-^13^C_12_-lactulose and U-^13^C_6_-rhamnose (Cambridge Isotope Laboratories), as internal standards (ISs). The samples were extracted and derivatized using N, O-bis[TMS]trifluoroacetamide containing 1% trimethylchorosilane (Sigma Aldrich) and analyzed by GC-MS (6890 N/5973B Agilent Technologies) monitoring ions at m/*z* 361 and 367 for L and 117 and 119 for R.

The percentage recovery (%) of each sugar in the urine and the LR ratio, an index of gut mucosal integrity, were calculated as follows:

$$\begin{eqnarray*}
&& {\rm Percent\,\, lactulose\,\, or\,\, rhamnose\,\, recovery}\,\,(\%)\nonumber\\&&\quad = \left[({\rm Urine\,\, volume }({\rm mL})\,\,{\rm over\,\, first\,\, 2\,h}\right.\nonumber\\&&\qquad\left. \times\, {\rm lactulose\, or\, rhamnose\,\, concentration\,\,}({\rm \mu\, mol/L})\,\,{\rm in}\,\, {\rm urine})/\right.\nonumber\\&&\quad\qquad\left. {\rm dose\,\, of\,\, lactulose\,\, or\,\, rhamnose\,\, administered} \right] \times\! {\rm{ 100}}
\end{eqnarray*}$$

The LRR was calculated as the ratio of L (%) and R (%) recovery.

After completion of the LRR measurement protocol, a single blood sample was collected (∼4 h postprandially, which was considered as a fasting sample), which was used to measure plasma Trp and Kyn concentrations (to calculate KTR), as well as baseline isotopic enrichment for the tracer protocol to measure Trp kinetics. This sample was also used to measure plasma Ala, cytokines, and basic blood parameters such as hemoglobin and red and white blood cell counts (ABX-Pentra 60C+, Horiba Medical).

### Trp tracer kinetic protocol

**FIGURE 1:**
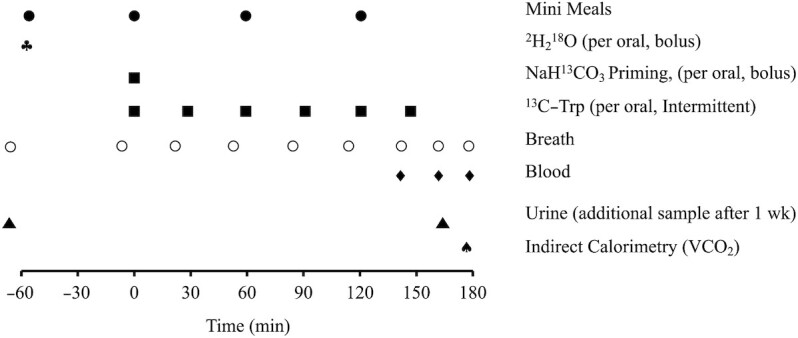
The ^13^C_1_-tryptophan isotope tracer protocol along with double-labeled water dosing.

A typical experimental day started at ∼09:00, with the arrival of children accompanied by their mothers to the metabolic ward at St John's Medical College. This experimental protocol was conducted within a period of 1 wk from the screening (LR protocol). The mothers were instructed to provide an early morning breakfast to their children and to avoid adding cane sugar to their meals from the previous day (to reduce variation of ^13^C abundance in the bicarbonate/CO_2_ pool). A 2-h postprandial period was ensured before starting the tracer experiment. At −60 min, and 5 min before dosing, a baseline breath sample was collected. From 0 min onward, a standard meal of a cereal–legume mix (khichdi), proportional to body weight (BW), was administered hourly in 4 equal portions for 4 h. After the first-hour meal, oral ^13^C-Trp, 99%, (Cambridge Isotope Laboratories) was administered at one-half–hour intervals, at a dose of 4 µmol/kg BW/h (**Figure 1**). An initial dose of NaH^13^CO_3_ (3 µmol/kg BW, >99%, Cambridge Isotope Laboratories) was administered to prime the body bicarbonate pool and achieve early steady-state isotopic enrichment in breath CO_2_. An indwelling venous catheter (Jelco 24 G, Medex Medical Ltd.) was secured ∼120 min after the start of ^13^C-Trp dosing, to collect 3 sequential blood samples at 15-min intervals, from 150 min onward (at 150, 165, and 180 min), which reflected the steady-state period. Postdose breath samples were collected immediately before consumption of every half-hourly ^13^C-Trp dose until 150 min of the protocol, and then every 15 min until the end of the protocol. A ventilated hood (5-L capacity)–based indirect calorimetry measurement of CO_2_ excretion (VCO_2_) was performed between 150 and 180 min of the protocol, while the child slept with minimal or no movements. This system was calibrated by ethanol combustion, with an error of <2% for measurement of VCO_2_.

### Breath sample collection and analysis

Breath samples were collected using a mask (covering the nose and mouth of the child) connected with a 1-way valve into a nondiffusible disposable bag, allowing only expired breath to be collected. After each collection the samples were transferred into two 10-mL non–silicon-coated tubes (Vacutainer; Becton Dickinson) and stored at room temperature until analysis. The ^13^CO_2_ abundance (atom %) in the breath samples was analyzed using an isotope ratio mass spectrometer (IRMS, Delta V Advantage, Thermo Fisher Scientific Inc.). The increase in ^13^CO_2_ enrichment during the fed state of the tracer protocol was expressed as atom percent excess over baseline abundance. The precision of the IRMS for CO_2_ measurement was <0.006%.

### Blood collection and analysis

Blood samples collected during screening (basal sample), and the Trp tracer protocols were used to measure basal (or fasted) plasma Trp, Kyn, and amino acid (AA) concentrations, as well as fed-state plasma Trp and Kyn concentrations and ^13^C enrichments of Trp, Kyn, and Ala. Blood was collected into EDTA vacutainers (Becton Dickinson), centrifuged at 1098 x g for 10 min at 4^⁰^Cand the plasma was divided into aliquots that were placed into sterile cryovials and stored at −80^⁰^C until analysis. Dansylated (DANS) derivatives of plasma Trp, Kyn, and Ala were analyzed by LC-MS/MS (TSQ Vantage, Thermo Scientific) monitoring the following precursor and product ions: for DANS-Trp, ions m/z 439 and 170 (from ^13^C-Trp tracer), for DANS-Kyn, m/z 443 and 170 (from ^13^C-Trp tracer) and for DANS-Ala, m/z 324 and 170 (from ^13^C-Trp tracer).

Screening plasma and postdose plasma from the tryptophan tracer kinetics experiment, were used to measure the Trp and Kyn concentrations by an isotope dilution method. Briefly, a known amount of plasma sample was spiked with a known quantity of indole-^2^H_5_-tryptophan and ^2^H_6_-kynurenine (Cambridge Isotope Laboratories) as IS. The samples were analyzed by LC-MS/MS by monitoring the following precursor and product ions: 443 and 170 (from ^2^H_5_-Trp IS) and 448 and 170 (from ^2^H_6_-Kyn IS). Plasma AA concentrations were measured by ultra-performance LC (ACQUITY H-Class System, Waters Corporation) using precolumn derivatization with 6-aminoquinolyl-N-hydroxysuccinimidyl carbamate (Waters AccQ⋅Tag™ assay kit) with norvaline (Sigma Aldrich) as IS. Plasma samples were deproteinized with 10% sulfosalicylic acid dihydrate, and derivatized using AccQ-Fluor™ derivative reagent. The derivatized AAs were separated using a gradient-based ACQUITY UPLC BEH C18 column (130 Å, 1.7 µM, 2.1 mm × 150 mm) with an ACQUIT UPLC Tunable UV (TUV) detector.

### Calculations for Trp kinetics

All kinetic data were expressed per unit of FFM. The calculations were performed using standard equations as previously described (24).

The total flux (*Q*) of tryptophan was calculated from the standard steady-state equation:

(1) $$\begin{eqnarray*}
Q\,\,(\mu\,\, mol/kg\,\,FFM/h) = ( {{\rm E}_{\rm inf}}\,\,/\,\,{{\rm E}_{\rm plat}}) \times {\rm i}
\end{eqnarray*}$$

where E_inf_ is the isotopic enrichment of Trp in the infusate, E_plat_ is the isotopic enrichment of Trp in plasma at isotopic steady state, and “*i*” is the infusion rate of the tracer (µmol/kg FFM/h). Endogenous flux is equal to total flux minus intake from the infusion plus diet in the fed state.

The percentage of administered ^13^C-Trp that was oxidized was calculated from the following equation:

(2) $$\begin{eqnarray*}
Trp\,\,Oxd\,\, (\%) = \left[({{\rm Ra\,\, CO}_{\rm 2}}\,\, \times {{\rm E\,\, CO}_{\rm 2}}) / {\rm i}_{\rm Trp} \right] \times {\rm 100}
\end{eqnarray*}$$

where Ra CO_2_ = VCO_2_/0.82, the rate of CO_2_ excretion divided by 0.82, to correct for the incomplete excretion of CO_2_ produced in the fed state; E CO_2_ is the plateau isotopic enrichment of breath CO_2_; and i_Trp_ is the rate of administration of the oral ^13^C-tryptophan tracer.

Total Trp oxidized was calculated according to the following equation:

(3) $$\begin{eqnarray*}
&& Total\,\,Trp\,\,Oxd\,\,({\mu mol/kg\,\, FFM/h}) \nonumber\\&&\quad = {\rm Trp\,\, flux} \times {\rm \%\,\, Trp\,\, oxidized}
\end{eqnarray*}$$

Nonoxidative flux of Trp, an index of Trp bioavailability for total protein synthesis, was calculated as follows:

(4) $$\begin{eqnarray*}
{\rm Trp\,\, flux\,\, to\,\, Protein\,\, Synthesis} = {\rm Eq 1 - Eq 3}
\end{eqnarray*}$$

The fractional synthesis rates (FSRs) of Kyn or Ala from Trp were calculated according to the precursor–product equation:

(5) $$\begin{eqnarray*}
FSR\,\,(\%\,\, pool/h) &=& \left[ ({{\rm Eprod}_{\rm t2}} - {{\rm Eprod}_{\rm t1}})/{\rm Epre} \right]\nonumber\\&&\times ({\rm 24 \times 100})/({{\rm t}_{\rm 2}} - {{\rm t}_{\rm 1}})
\end{eqnarray*}$$

Where Eprod_t2_ – Eprod_t1_ was the increase in isotopic enrichment of the product (Kyn or Ala) over the time interval (t_2_ − t_1_) of the infusion and Epre was the plateau isotopic enrichment in plasma of the precursor, Trp. The absolute (total) synthesis rate of plasma Kyn or Ala, that is, the rate of conversion of Trp to Kyn or Ala, was calculated as the product of the Kyn concentration and its FSR, expressed as micromoles per liter of plasma per hour. The fraction of Kyn or Ala from Trp after 3 h was calculated as a ratio of Eprod and Epre.

### Urine collection, analysis, and calculation of body composition and total energy expenditure

The early morning urine voids of the children were collected at their homes and served as the basal sample for the tracer-based measurements of body composition and total energy expenditure (TEE). When the subjects arrived at the metabolic ward, they were administered double-labeled water (DLW) orally in a dose of 0.2 g/kg BW of 99% ^2^H and 3 g/kg BW of 10% ^18^O (Sercon Ltd.). A urine sample was collected between 3 to 4 h after dosing with DLW for body composition, and another urine sample was obtained after 1 wk for the estimation of TEE (Figure 1). The abundances of ^2^H and ^18^O in urine were measured in duplicate using a Delta V advantage IRMS (Thermo Fisher Scientific Inc.), with a precision of <1% for ^2^H and <0.08% for ^18^O.

Total body water (TBW) was estimated by the deuterium dilution method using ^2^H enrichment after dosing, in the standardized protocol per the International Atomic Energy Agency recommendation (25). Fat free mass (FFM) was calculated as TBW/*F*, where *F* was an age- and sex-specific hydration constant; fat mass (FM) was then calculated as the difference between BW and FFM.

TEE was calculated from urinary water ^2^H and ^18^O enrichment as follows: the elimination rate (Rx) of each isotope was calculated from the slope of the natural log of the enrichment plotted against time: Rx = ln (E2/E1)/(t2 – t1), where E was the enrichment (parts per million) and t is time (days) after dose administration, and x was either ^2^H or ^18^O. As ^2^H is eliminated from the body as water, whereas ^18^O is eliminated as water plus carbon dioxide, the difference between the elimination rates of the 2 isotopes gives the carbon dioxide production rate, which is used to calculate TEE using standard indirect calorimetric equations (24).

### Fecal collection, inflammatory biomarkers, and cytokine analysis

A fecal sample was collected by the mother at home, who was instructed to use sterile gloves and container, 1 wk after the experiment. The fecal sample was transferred immediately in an icebox from the participant's home and stored at −20^⁰^C until further analysis. Fecal myeloperoxidase (EDI Epitope Diagnostics Inc.), neopterin (Demeditec Diagnostics GmbH), α-1 anti-trypsin (ImmuChrom GmbH), and calprotectin (EDI Epitope Diagnostics Inc.) was measured in duplicates by using standardized ELISA kits, with precision rates of 13, 15, 12, and 8%, respectively. Basal plasma samples (only *n* = 19 were available) were used to measure 13 cytokines [granulocyte-macrophage–colony-stimulating factor, IFN-γ, IL10, IL-12, IL-13, IL-1β, IL-2, IL-4 to IL-8, and TNF-α] using an ELISA-based 13-Plex cytokine assay (Milliplex map kit, EMD Millipore Corporation), with a precision, for all cytokines, ranging between 0 and 5%.

### Statistical analysis

In the absence of previously published data related to the primary study objective, a sample size of 16 children per group was sufficient to observe a 10% difference, between the EED and non-EED groups, in Trp oxidation or Trp oral availability, with an assumed SD of 10%, power of 80%, and level of significance 5%. To account for a 20% dropout rate, a total of 40 participants were recruited. The normality of all measured variables was examined using Q-Q plots. SES indicators were combined to derive the water and sanitation, assets, maternal education, and income (WAMI) index, which was used as a continuous variable in association analyses. The usual dietary intake of study participants from the slum community was adjusted to partially remove the day-to-day variability in intakes (within-person variation). Statistical adjustments for within-person variability in nutrient intake were performed (26). Group differences for demographic, anthropometric, EED biomarkers (Trp, Kyn concentration, KTR, and fecal biomarkers of gut inflammation) and Trp kinetic parameters were determined by using the Mann-Whitney U test. The difference in TEE between EED groups, adjusting for FFM, was examined using linear regression of TEE. A mixed linear model was used to examine association of KTR with state (fasted compared with fed, paired observations), EED groups, and the interaction between the 2, with subject as the random effect.

Since EED could present in varying degrees of severity (as a continuum) in children from the slum, continuous associations between EED variables were also examined. Correlations between Kyn parameters (fasted plasma Trp, Kyn concentration, and KTR) and WAMI index, plasma citrulline concentration (as a marker of enterocyte mass), and markers of inflammation (plasma cytokines, fecal biomarkers), along with anthropometric indices, were examined using Spearman's rank correlation coefficient, to confirm established links between different EED descriptor domains (27). Continuous correlations were also evaluated between the determinants of TDO (high leucine intake) or IDO (IFN-γ as the principal effector, with other inflammatory cytokines like IL-6, TNF-α, IL-1β) induction and fasted plasma Kyn and Trp concentrations, KT, Trp oxidation, and fraction of Ala from Trp after 3 h, using Spearman's rank correlation coefficient. Statistical significance was considered at *P* < 0.05. Analysis was performed using STATA (StataCorp. 2019. Stata Statistical Software: Release 16; StataCorp LP).

## Results

Demographic, anthropometric, and nutrient intakes (albeit from a single-day recall) of children from the high-SES community (control group) are provided in **Table 1**. The high-SES children had normal nutritional status indicators, with median HAZ, WAZ, and WHZ > −1 *z*-score. Based on the LRR values obtained for the 20 healthy high-SES children, the upper limit of normal (mean + 2 SDs) for LRR was calculated to be 0.068, and this cutoff value was used to designate 2 groups of the slum children: the EED group (LRR ≥ 0.068) and the no-EED group (LRR < 0.068). Demographic, anthropometric, and EED parameters, and nutrient intakes of children from the slums, categorized as no-EED and EED, are also provided in Table 1. Children categorized as EED had a significantly lower WAMI index and were shorter and lighter, with lower FFM (but not as percentage of BW), and were likely to be stunted and underweight when compared with those with no-EED. There was no difference in fecal biomarker concentrations between the groups. The fasted plasma concentrations of asparagine (*P* = 0.041) and glycine (*P* = 0.036) were significantly lower, and the plasma concentration of citrulline tended toward significance (*P* = 0.054) in the EED compared with the no-EED group. The median (quartile 1, quartile 3) concentrations (µmol/L) of asparagine, glycine, and citrulline were 22.3 (19.5, 31.9), 173.5 (151.2, 200.9), and 34.0 (20.1, 26.9) in the EED group compared with 30.3 (24.1, 41.3), 198.2 (179.0, 229.3), and 27.1 (23.0, 34.4) in the no-EED group (**Supplemental Table 1**). The FFM-adjusted TEE (kcal) was significantly higher in the EED group (*β*:118, 95% CI: 21, 215). There was no difference in nutrient intakes between the groups.

**TABLE 1:** Demographic, anthropometric, and environmental enteric dysfunction (EED)-related characteristics of children from a high-SES community (control group) and those from a low-SES community (test group), categorized as EED or no-EED based on LRR^1^

	High-SES control	^2^No-EED (LRR <0.068)	^2^EED (LRR ≥0.068)	^3^ *P* value
*n*	20	19	27	
Sex, F/M	8/12	9/10	15/12	
Age, mo	21.1 (18.0, 24.5)	21.2 (20.0, 22.9)	20.7 (18.8, 23.7)	0.902
Maternal education, y	15 (12, 15)	10 (10, 10)	9 (3, 10)	0.192
WAMI index	0.92 (0.87, 0.95)	0.72 (0.69, 0.75)	0.64 (0.50, 0.73)	0.011
Hemoglobin,^4^ g/dL	—	9.3 (8.3, 10.0)	9.1 (8.1, 10.4)	0.953
CRP,^4^ mg/L	—	0.2 (0.1, 0.7)	0.2 (0.2, 0.3)	0.389
Anthropometry				
Height, cm	83.0 (78.2, 85.2)	80.3 (79.0, 83.6)	78.5 (75.0, 80.1)	0.010
Weight, kg	10.5 (9.7, 11.1)	9.9 (9.4, 10.6)	8.9 (8.4, 9.6)	0.005
FFM, kg	—	7.1 (6.7, 8.2)	6.4 (6.2, 7.2)	0.010
FFM, % BW	—	73.3 (69.5, 77.2)	74.2 (71.2, 77.9)	0.882
Total energy expenditure, kcal/d^4^	—	736.5 (589.9, 881.8)	784.4 (745.6, 835.6)	0.312
HAZ	−0.6 (−1.0, −0.0)	−1.4 (−2.0, −0.7)	−2.0 (−2.6, −1.6)	0.005
WAZ	−0.5 (−1.1, −0.2)	−1.1 (−1.9, −0.7)	−1.9 (−2.3, −1.6)	0.004
WHZ	−0.4 (−0.7, 0.1)	−1.0 (−1.3, −0.4)	−1.2 (−1.6, −0.8)	0.090
EED parameters				
Dual sugar assay				
Rhamnose recovery, %	2.80 (2.40, 4.61)	2.19 (1.45, 3.84)	1.37 (0.83,2.56)	0.035
Lactulose recovery, %	0.065 (0.044, 0.114)	0.115 (0.070, 0.166)	0.168 (0.116, 0.395)	0.028
LRR	0.019 (0.014, 0.046)	0.048 (0.036, 0.059)	0.097 (0.078, 0.173)	<0.001
Fecal biomarkers^4^				
MPO, ng/mL	—	59,221 (43,233, 80,226)	71,668 (50,815, 95,192)	0.204
NEO, nmol/L	—	1151 (791, 2849)	1107 (728, 2362)	0.599
AAT, mg/g	—	0.53 (0.19, 0.97)	0.59 (0.38, 0.80)	0.748
Calprotectin, µg/g	—	135 (91, 490)	244 (112, 740)	0.287
Nutrient intakes				
Energy, kcal/d	1080 (916, 1265)	1037 (866, 1247)	948 (843, 1129)	0.200
Protein, g/d	29 (25, 37)	29 (21, 37)	29 (23, 33)	0.815
Fat, g/d	31 (27, 42)	30 (22, 45)	28 (22, 37)	0.403
Tryptophan, mg/d	352 (377, 448)	338 (257, 403)	335 (266, 394)	0.647
Leucine, g/d	2.4 (1.9, 3.0)	2.3 (1.7, 3.1)	2.3 (1.9, 2.8)	0.729
Niacin, mg/d	4.5 (3.6, 6.2)	5.2 (3.9, 5.5)	5.0 (4.0, 6.0)	0.902

1 Values are median (quartile 1, quartile 3) unless otherwise indicated. AAT, α-1 antitrypsin; CRP, C-reactive protein; FFM, fat-free mass; HAZ, height-for-age *z*-score; LRR, lactulose rhamnose ratio; MPO, myeloperoxidase; NEO, neopterin; SES, socioeconomic status; WAMI, water, sanitation, assets, maternal education, and income index; WAZ, weight-for-age *z*-score; WHZ, weight-for-height *z*-score.

2 No-EED is defined as LR ratio < 0.068 and EED as ≥ 0.068; the mean + 2 SD (0.029 ± 0.019) of LRR (0.068) from controls was used as the upper limit of normal.

3 *P* value for Mann-Whitney U test between the EED groups

4 Hemoglobin *(n* = 19 and 25); CRP *(n* = 13 and 22), Total energy expenditure *(n* = 18 and 22), Fecal biomarkers *n* (17 and 25) for no-EED and EED, respectively. These data were not available in all children due to lack of blood, urine, or stool sample.

The plasma concentrations of Trp and Kyn along with the KTRs of children with and without EED in the fasted (∼4 h postprandially) and fed states are presented in **Table 2**. The median (quartile 1, quartile 3) fasted KTR was 0.089 (0.066, 0.110) in children with EED compared with 0.070 (0.050, 0.093) in children with no-EED (*P* = 0.077). The fasted KTR seemed to be driven by the association of Trp with KTR (*ρ*s = −0.87, *P* < 0.001) rather than that of Kyn (*ρ*s = −0.41, *P* = 0.005). The fed-state KTR was not different between the groups (*P* = 0.613). However, when KTR was compared between EED categories, with both fasted- and fed-state data combined in a mixed model, there was a significant difference between the 2 groups such that the KTR was higher in the EED group (*β*: 0.0203; 95% CI: 0.0002, 0.0406; *P* = 0.048).

**TABLE 2:** Plasma concentrations of Tryptophan (Trp) and Kynurenine (Kyn) and the Kyn to Trp ratio (KTR) of children with and without environmental enteric dysfunction (EED) in the fed and fasted states^1^

	No-EED (LRR <0.068)	EED (LRR ≥0.068)	*P* value^2^
*n*	19	26	
Fasted state			
Trp concentration, µmol/L	44.5 (34.4, 63.9)	39.1 (26.8, 56.7)	0.323
Kyn concentration, µmol/L	3.2 (2.7, 3.8)	3.6 (2.8, 4.1)	0.228
KTR	0.070 (0.050, 0.093)	0.089 (0.066, 0.110)	0.077
Fed state			
Trp concentration, µmol/L	51.5 (45.3, 61.5)	48.6 (41.7, 60.8)	0.260
Kyn concentration, µmol/L	3.8 (3.0, 5.4)	3.9 (3.4, 5.0)	0.982
KTR	0.078 (0.051, 0.106)	0.079 (0.069, 0.108)	0.613

1 Values are median (quartile 1, quartile 3). LRR, lactulose rhamnose ratio.

2 *P* value for Mann-Whitney U test between the EED groups

The Trp kinetics data are presented in **Table 3**. There was no difference in the total fed-state Trp oxidation (overall, a median value of 7.4% of oral Trp intake was oxidized) or Trp availability for protein synthesis between the EED and no-EED groups. The median (quartile 1, quartile 3) dietary Trp intake was 36 (28, 42) mg/kg/d, which was well in excess of the maximum possible daily irreversible Trp oxidation rate, based on an extrapolation of the hourly fed-state oxidation rate to 24 h. The fractional and absolute synthesis ratesof Kyn and the fraction of Ala derived from Trp after 3 h in the plasma compartment were significantly slower in the EED group (Table 3).

**TABLE 3:** Whole-body tryptophan (Trp) kinetics and rate of synthesis of kynurenine (Kyn) and alanine (Ala) from Trp in children with and without environmental enteric dysfunction (EED) in the fed state^1^

n	No-EED (LRR <0.068) (*n* = 19)	EED (LRR ≥0.068) (*n* = 26)	*P* value^2^
Total Oral Trp intake, µmol/kg FFM/h	13.6 (12.3, 14.2)	14.1 (13.4, 14.6)	0.166
Total Trp flux, µmol/kg FFM/h	47.6 (39.0, 50.6)	46.7 (40.7, 51.9)	0.597
Total Trp oxidized,^3^ µmol/kg FFM/h	3.9 (1.8, 6.0)	3.1 (1.3, 5.8)	0.617
Oral Trp oxidized,^3^ %	7.9 (4.1, 13.1)	6.6 (3.3, 13.2)	0.639
Oral Trp oxidized,^3^ µmol/kg FFM/h	1.0 (0.6, 1.8)	0.9 (0.4, 1.7)	0.868
Index of whole-body protein breakdown			
Endogenous Trp flux, µmol/kg FFM/h	34.0 (25.2, 37.0)	32.9 (27.1, 38.1)	0.679
Index of whole-body protein synthesis			
Nonoxidative Trp disposal,^3^ µmol/kg FFM/h	42.5 (37.9, 46.9)	42.6 (36.5, 45.7)	0.868
Bioavailability of oral Trp			
Oral Trp balance,^3^ µmol/kg FFM/h	12.4 (10.8, 13.9)	12.4 (11.9, 13.7)	0.575
Rate of Kyn and Ala synthesis			
Kyn synthesis from Trp			
FSR, %pool/h	21.3 (16.1, 24.7)	12.5 (5.4, 20.0)	0.005
Absolute synthesis rate, µmol/L plasma/h	0.81 (0.54, 1.15)	0.46 (0.21, 0.75)	0.004
Fraction of Kyn from Trp after 3 h	0.58 (0.52, 0.65)	0.53 (0.45, 0.59)	0.073
Ala synthesis from Trp			
FSR, %pool/h	1.6 (0.6, 2.5)	1.0 (0.4, 2.2)	0.491
Absolute synthesis rate, µmol/L plasma/h	4.4 (1.6, 9.9)	3.8 (1.2, 7.9)	0.505
Fraction of Ala from Trp after 3 h	0.012 (0.008, 0.018)	0.007 (0.005, 0.015)	0.037

1 Values are median (quartile 1, quartile 3) unless otherwise indicated. FFM, fat free mass; FSR, fractional synthesis rate; LRR, lactulose rhamnose ratio.

2 *P* value for Mann-Whitney U test between the EED groups.

3 *n* = 16 and 21 for no-EED and EED, respectively. In 3 and 4 children from the no-EED and EED groups, respectively, the breath ^13^CO_2_ abundance did not increase.

Fasted plasma Kyn concentration was significantly and positively correlated with leucine intake (*ρ*s = 0.31, *P* = 0.046) and fasted leucine concentration (*ρ*s = 0.52, *P* = 0.001), whereas fasted plasma Trp concentration was inversely associated with IL-1β (*ρ*s = −0.54, *P* = 0.016). Total Trp oxidation (µmol/kg FFM/h) significantly and positively correlated with fed state plasma leucine (*ρ*s  = 0.34, *P* = 0.037), valine (*ρ*s  = 0.38, *P* = 0.021) and IFN-γ (*ρ*s  = 0.57, *P* = 0.022). The fraction of Ala from Trp after 3 h significantly and positively correlated with fasted and fed plasma Trp concentration (*ρ*s  = 0.44, *P* = 0.002 and *ρ*s  = 0.50, *P* = <0.001, respectively), fecal neopterin (*ρ*s = 0.42, *P* = 0.006), and inversely correlated with IFN-γ (*ρ*s = −0.46, *P* = 0.050) and leucine (*ρ*s = −0.30, *P* = 0.046) and niacin (r_s_*ρ*s = −0.29, *P* = 0.050) intake. The cytokine concentrations for all children (*n* = 19) are provided in **Supplemental Table 2**.

## Discussion

This study showed that the EED group tended (*P* = 0.077) to have a higher fasting plasma KTR, than the no-EED group, as expected. When KTR was compared between EED categories, with both fasted- and fed-state data combined in a mixed model, there was a significant difference between the 2 groups such that the KTR was higher in the EED group. The interpretation of a high KTR is that there is a greater diversion of Trp to Kyn, leaving less Trp available for body protein synthesis and hence growth. However, when Trp kinetics were measured by a tracer protocol, there were no differences between the EED and no-EED groups in Trp oxidation or Trp availability (measured as the nonoxidative Trp disposal rate) for whole-body protein synthesis. Contrarily, the EED group had a significantly slower rate of Trp conversion to Kyn and Ala than the no-EED group. These findings do not support the hypothesis that there is accelerated Trp catabolism to Kyn in the EED group as a result of intestinal or systemic inflammation (14,15), even though this group had high (static) plasma KTR concentrations.

**FIGURE 2:**
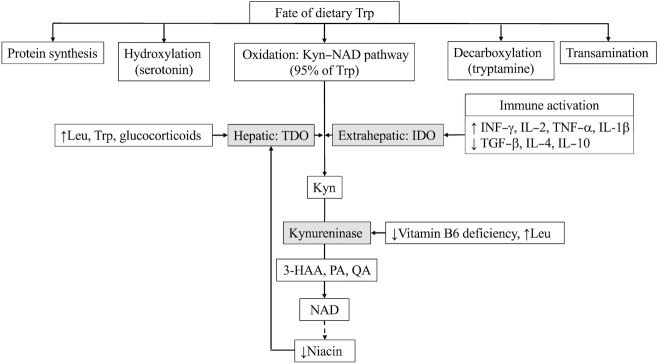
Fate of dietary Trp and regulation of the kynurenine (Kyn)–NAD pathway. Trp can become limiting for protein synthesis if the oxidation pathway is accelerated, which in turn affects growth. Kyn plasma concentration increases when Trp oxidation exceeds renal handling. Niacin accumulation inhibits the pathway in a negative feedback fashion, whereas the need for more (for example, dietary deficiency), drives Trp down the oxidative pathway. Enzymes, gray-shaded boxes; ↑, upregulates the enzyme; ↓, downregulates the enzyme. IDO, indoleamine 2,3-dioxygenase; Kyn, kynurenine; Leu, leucine; PA, picolinic acid; QA, quinolinic acid; TDO, tryptophan 2,3-dioxygenase; TGF, transforming growth factor; Trp, tryptophan; 3-HAA,  3-hydroxy anthranilic acid.

There are alternative hypotheses to explain these paradoxical findings (**Figure 2**). First, both EED and no-EED children came from the same environmentally stressed area, with poor sanitation and hygiene. Hence, the probability of a variable degree of EED in all children cannot be ruled out. The increased 3-h production rate of Kyn and Ala from Trp in the no-EED group is an indicator of the higher flux of Trp further down the Trp–Kyn pathway, producing important downstream protective metabolites, such as 3-hydroxy anthranilic acid (3-HAA), picolinic acid, quinolinic acid (QA), and NAD, which could dampen the damaging effect of proinflammatory cytokines on the intestinal mucosa (28). For example, QA and 3-HAA induce apoptosis in T-helper type 1 cells, and thereby could control CD4 + T-cell–induced intestinal inflammation and increased permeability (28). A limitation to this reasoning is that the generation of these downstream metabolites of the Kyn–NAD pathway were not measured in the present study. Nevertheless, this brings to the fore the issue of a dynamic nature of the pathophysiology of EED, whereby a group of children with possible EED might have normal permeability markers because of successful protective adaptations in other pathways. Second, the Trp–Kyn conversion rate could merely be an indicator of higher activity of the constitutive hepatic TDO (and not IDO) in the no-EED group. TDO induction depends on the free (unbound) Trp concentration, or free cellular leucine or cortisol concentrations. Further, the induction of TDO could also occur in order to increase Trp catabolism to niacin to meet that daily requirement (Figure 2). The present study, however, showed no difference between the EED and no-EED groups in their fed-state plasma leucine or Trp concentration, or in leucine, Trp, or niacin intakes. Third, in the EED group, it is possible that Trp was being selectively partitioned for protein synthesis due to low Trp availability as a result of either poor digestion of Trp or its microbial degradation in the gut, thereby reducing its availability for oxidation; as small intestinal bacterial overgrowth is a well-documented occurrence in EED (29, 30). In this study, it might be that a change in Trp handling through TDO induction is more plausible than an infective etiology, as the latter was not supported by differences in plasma C-reactive protein (CRP) concentration or fecal biomarkers between the groups. Furthermore, in a previous study we have shown that the KTR was strongly correlated (*n* = 18, *ρ*s = −0.77, *P* = 0.009) with leucine digestibility in healthy adults (31), emphasizing the relevance of leucine in Trp catabolism.

In effect, the KTR, a static plasma measurement, is probably not reflective of the evolving and adaptive nature of EED, or of the dynamic kinetics of Trp conversion, as Kyn is the first intermediary metabolite of a complex metabolic pathway. The KTR is dependent on the half-life of the Kyn pool as well, and if there is a greater modulatory demand for Kyn metabolites, as discussed above, then the KTR could be unchanged or even lower than anticipated in EED. Experimental evidence in animals supports this line of thought, as pigs housed in poor sanitary conditions with systemic inflammation showed no increase in Kyn plasma concentrations, despite induction of IDO activity, as measured by increased enzyme expression (13). Trp diversion to Kyn and its eventual oxidation did not limit its availability for protein synthesis, as the dietary Trp intake of 31 mg/kg/d, even after correcting for an assumed digestibility of 0.7 for a cereal-predominant diet, was well over the daily Trp requirement of 6.4 mg/kg/d for this age group (32).

An LRR cutoff of ≥0.068, obtained in this study from apparently healthy age- and sex-matched children belonging to a high SES, was used to group children recruited from urban slums into those with and without EED. This cutoff value coincides with the LRR cutoff from another study in young children (>0.07), below which presence of normal small intestinal morphology was reported (33). The prevalence of EED in children aged 18–24 mo in the present study was 59%, which is very similar to the 64% observed in children from urban slums of Bangladesh, whose median age was 15 mo (21). In agreement with this classification, children classified as having EED in this study had a poorer WAMI index and had a greater prevalence of stunting and were underweight compared with children in the no-EED group. They also tended to have a lower plasma citrulline concentration, which is synthesized exclusively in the small gut and is considered a reliable marker of enterocyte mass (34). In addition, the EED group had lower plasma asparagine and glycine (a conditionally essential AA) after a brief fast (∼4 h). These findings suggest either increased utilization or suboptimal availability for intestinal immune barrier function as both of these AAs have been shown to have intestinal cytoprotective, anti-inflammatory, and antiapoptotic effects plus beneficial modifications of the gut microbial community toward production of SCFAs and driving resistance against pathogenic bacteria (35–37). A low glycine supply could also limit collagen synthesis in these children (38).

The FFM-adjusted TEE was also significantly higher in the EED group, probably due to subclinical inflammation/immune activation. In support of this hypothesis, Amazonian Shuar forager–horticulturalist children, who are exposed to environmental pathogens, have been shown to have subclinical immune activation, with ∼25% higher resting energy expenditure than their counterparts from developed countries (United States/United Kingdom) (39). However, their TEE was similar to that of the reference populations, owing to tradeoffs by having lower activity energy expenditure despite their higher physical activity; which is likely explained by improved energy efficiency in Shuar children. The higher TEE observed in the children with EED in this study suggest that metabolic adaptations are probably yet to evolve to allow tradeoffs due to their younger age.

The significant associations between fasting state plasma Kyn concentration with dietary leucine intake and fasting state plasma leucine concentration suggest the induction of hepatic TDO by leucine (28). The plasma Trp concentration in the fasting state was inversely associated with the inflammatory cytokine IL-1β, which is implicated in inducing TDO or IDO (40). Previous studies in this area have not considered the leucine intake, but they have shown significant inverse correlations between Trp concentration and proinflammatory cytokines (IFN-γ, ΙL-6, IL-10, CRP) and fecal neopterin (15, 16). The continuous evaluation of the Trp oxidation rate also provided a similar mixed picture, showing positive associations with fed-state plasma leucine, valine, and IFN-γ. Finally, the fraction of Ala generated from Trp after 3 h was positively associated with plasma Trp concentration in the fed state, suggesting Trp flux down the oxidative pathway secondary to induction of TDO (28). The fraction of Ala generated from Trp after 3 h also negatively correlated with IFN-γ, dietary leucine, or niacin intake, which indicates the use of Trp for niacin synthesis. These findings may reflect either TDO or IDO induction and different regulation of Kyn and nicotinamide metabolism by the available Trp.

The strength of this study is that it is, to our knowledge, the first to investigate measurement of Trp kinetics, using stable isotope–labeled Trp as a tracer, in children with suspected EED, along with characterization of the factors that influence Trp metabolism. However, due to the loss of the Trp-^13^C atom in the initial steps of the Kyn-NAD pathway, the downstream conversions of the labeled metabolites could not be traced. The interpretation of the findings from this study should take into account the small sample size, another possible limitation of this study, which was much lower for the cytokine assay, but the availability of blood samples, complexity, and cost of measurements did not allow for a larger evaluation. Even though it has been widely accepted as a diagnostic test for EED (e.g., 22, 41), the use of LRR to classify children as EED may be another limitation, as it fails to capture the variable and dynamic pathological manifestations of EED. There is also the possibility of undiagnosed lactose intolerance or cow milk or other protein-sensitive enteropathies of varying degree in the children, which might affect the results. In conclusion, static estimates of plasma KTR, as an indicator of Trp oxidation by IDO, do not reflect the dynamic nature of Trp flux down its oxidative pathway, with multiple functionally relevant downstream metabolites. In the context of EED, this observation implies the need to use more sensitive kinetic measurements (when interpreting biomarker data) to explore the balance between deleterious and protective mechanisms.

## Supplementary Material

nqac171_Supplemental_File

## Data Availability

Data described in the manuscript, code book, and analytic code will be made available upon request to the corresponding author.

## Notes

Supported by a grant (award number: OPP1133329) from the Bill and Melinda Gates Foundation to FJ and AVK and a grant to FJ from the USDA/ARS (6250-51000-051-00D-1). The funders were not part of the design, implementation, analysis, and interpretation of the data.

AVK is an Associate Editor for *The American Journal of Clinical Nutrition* and played no role in the Journal's evaluation of the manuscript.

Supplemental Figure 1 and Supplemental Tables 1 and 2 are available from the “Supplementary data” link in the online posting of the article at https://academic.oup.com/ajcn/.

AVK and FJ are co-senior authors.

Abbreviations used: AA, amino acid; Ala, alanine; BW, body weight; CRP, C-reactive protein; DANS, dansylated; DLW, double-labeled water; EE, environmental enteropathy; EED, environmental enteric dysfunction; FFM, fat free mass; FM, fat mass; FSR, fractional synthesis rate; IDO1, indolamine 2,3-dioxygenase; IRMS, isotope ratio mass spectrometer; IS, internal standard; KTR, kynurenine tryptophan ratio; Kyn, kynurenine; Kyn-NAD, kynurenine nicotinamide di-nucleotide; L, lactulose; LAZ, length-for-age *z*-scores; LMIC, low-and-middle income countries; LRR, lactulose to rhamnose ratio; QA, quinolinic acid; R, rhamnose; ρ_s_, Spearman's rho; SES, socioeconomic status; TBW, total body water; TDO, tryptophan 2,3, dioxygenase; TEE, total energy expenditure; Trp, tryptophan; VCO_2_, volume of CO_2_ produced; WAMI, water and sanitation, assets, maternal education, and income; WAZ, weight-for-age *z*-scores; WLZ, weight-for-length *z*-scores; 3-HAA, 3-hydroxy anthranilic acid
