# Supplementary material for: Tryptophan oxidation in young children with environmental enteric dysfunction classified by the lactulose rhamnose ratio
Source: Am J Clin Nutr. 2022 Jun 14;116(4):970–9. doi: 10.1093/ajcn/nqac171 (PMC9535528; doi:10.1093/ajcn/nqac171)
Supplement: nqac171_Supplemental_File [file nqac171_supplemental_file.docx]

**Table 1**.  Plasma amino acid concentrations in children with and without environmental enteric dysfunction (EED) in the fasting and fed states^1^

| **Dispensable amino acids (µmol/L)** | | | | | | | |  | | | | | | | |
| --- | --- | --- | --- | --- | --- | --- | --- | --- | --- | --- | --- | --- | --- | --- | --- |
| ***Fasted State*** | Asn | Asp | Gln | Glu | Ser | Gly | Ala | | Arg | Cit | Orn | Pro | Tyr | Cys-Cys |  |
| No-EED^2^ (n=16)^3^ | 30.3  (24.1, 41.3)^4^ | 9.1  (7.4, 11.0) | 401.5  (354.4, 432.2) | 108.9  (82.0, 138.0) | 114  (103.6, 124.2) | 198.2  (179.0, 229.3)^4^ | 293.6  (250.8, 386.0) | | 78.3  (58.6, 101.8) | 27.1  (23.0, 34.3) | 39.1  (35.7, 45.3) | 163.6  (126.4, 189.8) | 54.9  (48.2, 61.1) |  |  |
| EED^2^ (n=24) | 22.3  (19.5, 31.9) | 9.1  (7.1, 12.3) | 415.8  (347.1, 468.0) | 121.3  (93.9, 133.0) | 115.9  (108.4, 127.2) | 173.5  (151.2, 200.9) | 269.8  (224.6, 303.3) | | 74.1  (45.3, 101.9) | 24.0  (20.1, 26.9) | 36.4  (30.0, 47.2) | 160.7  (143.4, 180.6) | 53.2  (47.0, 65.7) |  |  |
| ***Fed State*** |  |  |  |  |  |  |  | |  |  |  |  |  |  |  |
| No-EED (n=19) | 58.1  (49.7, 76.0) | 8.3  (7.3, 11.0) | 397.2  (373.3, 434.0) | 111.6  (86.7, 118.7) | 126.7  (116.8, 141.6) | 212.4  (177.0, 238.1) | 317.6  (268.3, 422.7) | | 93.4  (83.1, 119.7) | 24.7  (18.3, 30.6) | 54.4  (53.0, 62.6) | 162.6  (151.2, 199.8) | 76.1  (62.1, 84.9) | 1.0  (0.3, 1.5) |  |
| EED (n=26) | 56.7  (48.4, 73.9) | 8.3  (7.2, 10.3) | 398.5  (321.9, 485.0) | 107.3  (81.5, 136.7) | 128.6  (117.4, 146.9) | 189.4  (173.2, 240.4) | 361.4  (257.4, 409.2) | | 89.3  (76.6, 114.6) | 23.7  (19.1, 28.5) | 53.3  (41.6, 64.6) | 182.9  (142.7, 215.3) | 66.0  (58.2, 80.1) | 0.9  (0.5, 2.5) |  |
| **Indispensable amino acids (µmol/L)** | | | | | | | |  | | | | | | | |
| ***Fasted State*** | Phe | Lys | Met | Leu | Ile | Val | Thr | | His | Trp |  |  |  |  |  |
| No-EED (n=16) | 51.3  (47.8, 59.7) | 92.5  (78.9, 101.0) | 16.4  (14.5, 19.6) | 76.4  (68.2, 81.7) | 45.9  (41.2, 50.5) | 154.1  (145.7, 177.3) | 72.2  (54.5, 90.0) | | 71.3  (63.9, 78.3) | 34.7  (29.3, 41.3) |  |  |  |  |  |
| EED (n=24) | 53.2  (46.0, 60.9) | 103.9  (85.7, 122.9) | 17.0  (16.0, 19.8) | 77.3  (67.0, 108.1) | 50.3  (40.8, 60.0) | 151.0  (138.5, 211.9) | 67.0  (49.0, 83.7) | | 73.3  (66.4, 77.9) | 34.5  (26.7, 39.5) |  |  |  |  |  |
| ***Fed State*** |  |  |  |  |  |  |  | |  |  |  |  |  |  |  |
| No-EED (n=19) | 70.6  (66.5, 79.6 ) | 113.2  (104.2, 124.9) | 16.5  (14.9, 20.9) | 90.7  (74.3, 95.7) | 60.2  (56.4, 66.8) | 192.2  (166.4, 212.6) | 68.4  (60.6, 98.9) | | 75.5  (65.3, 82.0) | 48.3  (43.4, 56.2) |  |  |  |  |  |
| EED (n=26) | 68.0  (60.8, 78.1) | 117.2  (101.0, 147.1) | 18.0  (14.6, 19.3) | 88.6  (72.2, 99.3) | 60.0  (51.6, 64.6) | 181.3  (146.6, 201.1) | 69.9  (46.6, 88.9) | | 73.3  (68.7, 81.7) | 48.2  (40.9, 57.2) |  |  |  |  |  |

Abbreviations: Asn, Asparagine; Asp, Aspartic acid; Gln, Glutamine; Ser, Serine; Gly, Glycine; Ala, Alanine; Cit, Citrulline; Orn, Ornithine; Pro, Proline; Tyr, Tyrosine; Cys-Cys, Cysteine; Phe, Phenylalanine; Lys, Lysine; Met, Methionine; Leu, Leucine; Ile, Isoleucine; Val, Valine; Thr, Threonine; His, Histidine.

^1^Values are Median (Q1, Q3)

^2^No-EED is defined as LR ratio < 0.068 and EED as ≥ 0.068; the mean + 2SD (0.029 ± 0.019) of LRR (0.068) from controls was used as upper limit of normal

^3^These data were not available in all children due to lack of basal blood sample

^4^P value for Mann-Whitney U test between the EED groups <0.05

**Table 2**.  Plasma cytokine concentrations in the study children (n=19)^1^

| Cytokines | Concentration (pg/mL) |
| --- | --- |
| GM-CSF | 17.5 (10.9, 25.6) |
| INF-γ | 9.0 (6.9, 9.3) |
| IL10 | 9.4 (8.0, 11.5) |
| IL-12 | 2.1 (1.9, 2.2) |
| IL-13 | 5.7 (4.9, 6.6) |
| IL-1β | 0.8 (0.6, 0.9) |
| IL-2 | 1.2 (1.1, 1.4) |
| IL-4 | 40.6 (35.4, 42.3) |
| IL-5 | 2.1 (1.9, 2.7) |
| IL-6 | 2.2 (1.8, 2.7) |
| IL-7 | 9.6 (9.0, 10.3) |
| IL-8 | 5.7 (4.6, 11.3) |
| TNF-α | 9.5 (7.7, 10.9) |

Abbreviations: INF-γ, Interferon-γ; TNF-α, Tumour Necrosis Factor-α; IL, Interleukins.

^1^Values are Median (Q1, Q3)

**Supplemental Figure 1:** Subject screening and enrolment flowchart.

Assessed for eligibility from St. John’s Hospital (n= 93)

*High Socio-Economic Status*

Assessed for eligibility from urban slums

(n=384)

*Low Socio-Economic Status*

Enrolled (n= 21)

Enrolled (n=46)

Participated in lactulose rhamnose assay, to obtain a lactulose rhamnose ratio (LRR) cut-off to categorize slum children into those with environmental enteric dysfunction (EED) and those without (no-EED)

(n= 20)

Participated in lactulose rhamnose assay and *Tryptophan Tracer Kinetics*, *Body Composition and Total Energy Expenditure* protocol

(n= 46)

Completed both the studies and included for data analysis (n= 46)

Completed the studies and included for data analysis (n=20)

Excluded: refused to participate (n=72)

Excluded: refused to participate (n=338)

Excluded: not eligible, child stunted and underweight (n=1)
